# Supplementary figures and images for: Anethole inhibits human U87 Glioma cell proliferation by inducing apoptosis via the PI3K/AKT pathway
Source: PLoS One. 2025 Nov 21;20(11):e0336975. doi: 10.1371/journal.pone.0336975 (PMC12637905; doi:10.1371/journal.pone.0336975)

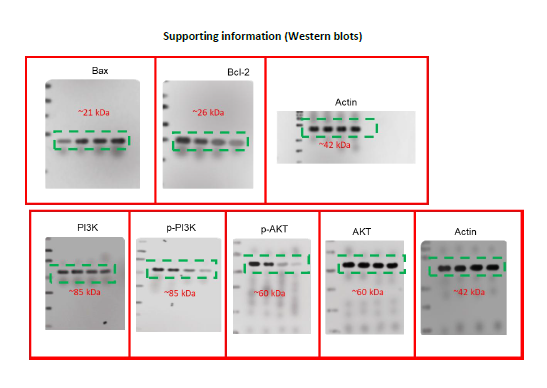


**Supplementary Figure S1. Uncropped blots with visible molecular weight markers**.

Supplement: S1 Fig — (DOCX) [file pone.0336975.s001.docx]
