# Supplementary material for: Anethole inhibits human U87 Glioma cell proliferation by inducing apoptosis via the PI3K/AKT pathway
Source: PLoS One. 2025 Nov 21;20(11):e0336975. doi: 10.1371/journal.pone.0336975 (PMC12637905; doi:10.1371/journal.pone.0336975)
Supplement: S2 File — Predicted physicochemical, pharmacokinetic, and drug-likeness properties of anethole were evaluated using the SwissADME web tool based on its SMILES structure. (DOCX) [file pone.0336975.s002.docx]

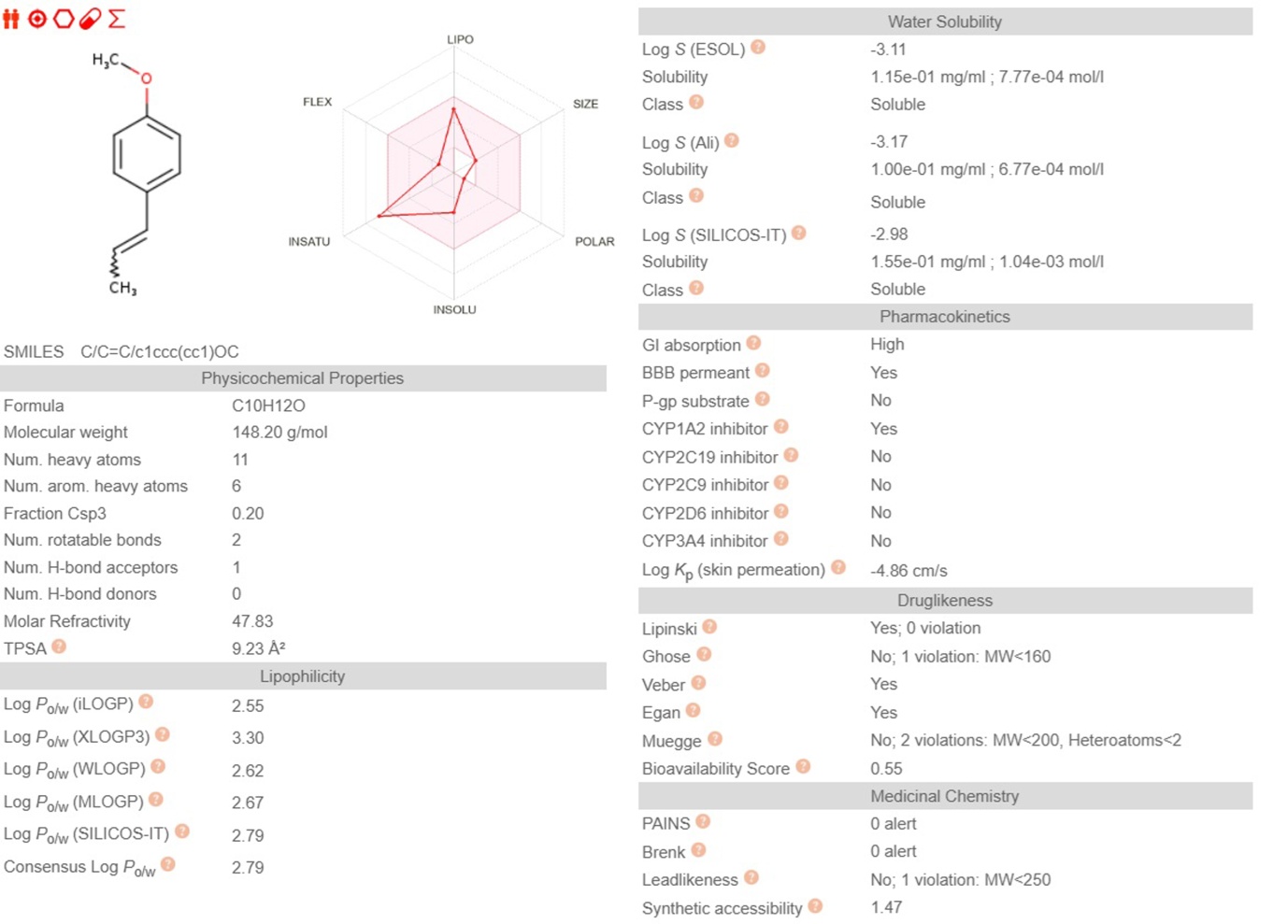


**Supplementary Figure S2.** **SwissADME analysis of anethole.** Predicted physicochemical, pharmacokinetic, and drug-likeness properties of anethole were evaluated using the SwissADME web tool based on its SMILES structure.
