# Supplementary material for: Anethole inhibits human U87 Glioma cell proliferation by inducing apoptosis via the PI3K/AKT pathway
Source: PLoS One. 2025 Nov 21;20(11):e0336975. doi: 10.1371/journal.pone.0336975 (PMC12637905; doi:10.1371/journal.pone.0336975)
Supplement: S3 File — The plot shows anethole’s predicted position within the yellow region, indicating blood–brain barrier (BBB) permeability. The white region represents high gastrointestinal absorption (HIA). The red circle (PGP–) marks anethole as a non-substrate of P-glycoprotein. (DOCX) [file pone.0336975.s003.docx]

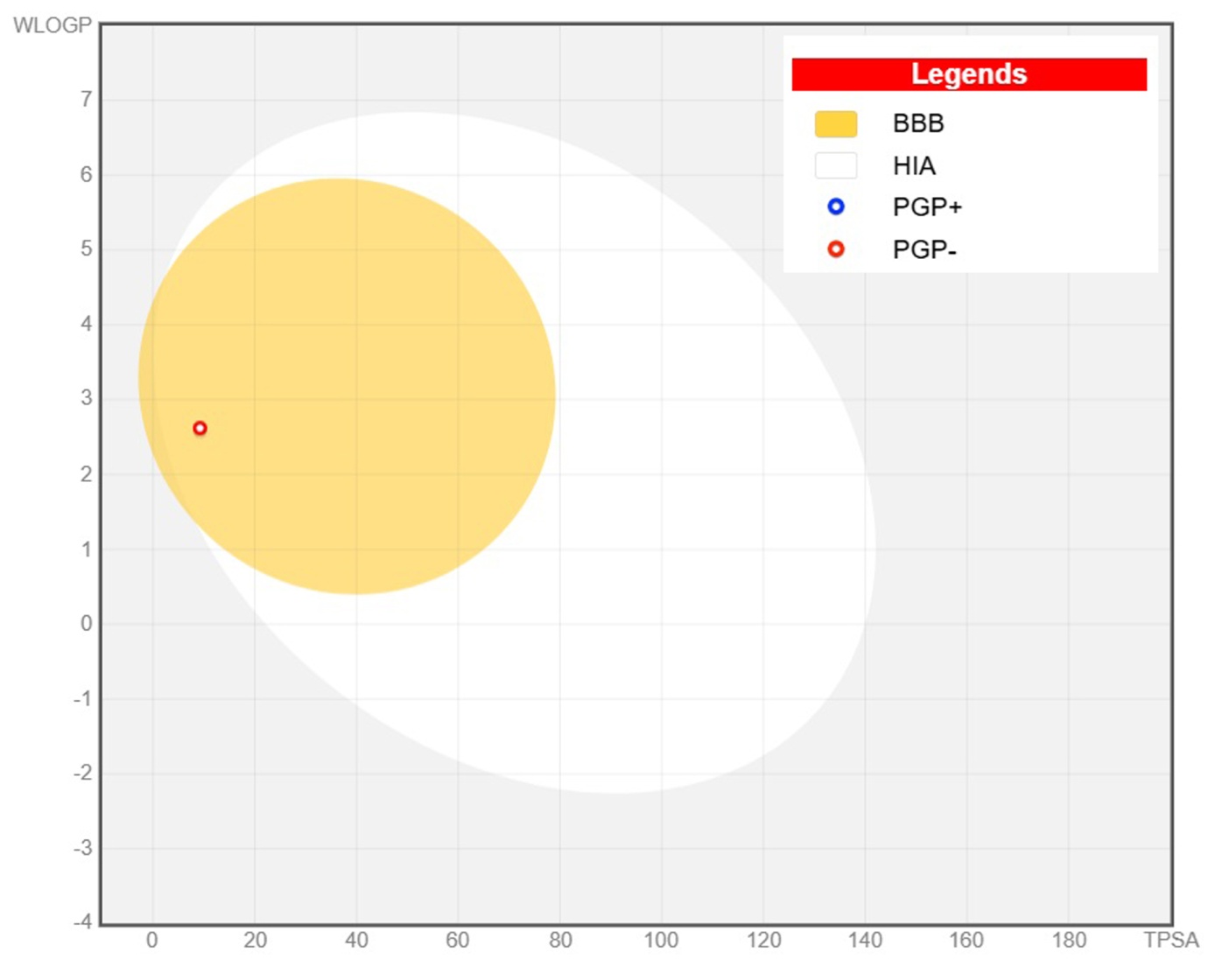


**Supplementary Figure S3.** **BOILED-Egg plot prediction o gastrointestinal absorption and brain penetration of anethole*.*** The plot shows anethole's predicted position within the yellow region, indicating blood–brain barrier (BBB) permeability. The white region represents high gastrointestinal absorption (HIA). The red circle (PGP–) marks anethole as a non-substrate of P-glycoprotein.
