# Supplementary material for: Anethole inhibits human U87 Glioma cell proliferation by inducing apoptosis via the PI3K/AKT pathway
Source: PLoS One. 2025 Nov 21;20(11):e0336975. doi: 10.1371/journal.pone.0336975 (PMC12637905; doi:10.1371/journal.pone.0336975)
Supplement: Attachment — Submitted filename: Response to reviewers.docx [file pone.0336975.s005.docx]

Dear editor,

We thank the reviewers for their thoughtful and constructive comments, which have significantly improved the quality and clarity of our manuscript. We have addressed each point below and made corresponding changes to the manuscript as indicated.

**Reviewer #1**

**Abstract**

1. **“Colony formation was notably inhibited in a dose-dependently.” → Grammatical error.**
   ✔ Corrected to “Colony formation was notably inhibited in a dose-dependent manner” in the abstract.
2. **How many replicates were performed for IC₅₀ determination? Mention explicitly.**
   ✔ We now state that all IC₅₀ values were calculated based on three independent experiments (n=3), with data expressed as mean ± standard deviation (SD).
3. **Western blot results should be summarized more concisely.**
   ✔ The Western blot summary in the abstract has been streamlined to focus on the major findings: induction of Bax and reduction of Bcl-2, and suppression of PI3K/Akt phosphorylation.

**Introduction**

4. **Page 2, Paragraph 2: Unclear phrasing regarding glioma malignancy.**
   ✔ Rephrased the sentence to: “Gliomas are essentially the most prevalent and primary brain tumors, constituting about 80% of all malignant intracranial neoplasms [1].”
5. **Page 3: "These findings underscore..." → Mention gaps in knowledge regarding anethole in gliomas.**
   ✔ Revised to note that although anethole shows promise in other cancers, its role in glioma remains unexplored and warrants targeted investigation. This has included in detail
6. **Provide a clearer rationale for targeting PI3K/Akt in glioblastoma.**
   ✔ We have added supporting context and references explaining that the PI3K/Akt pathway is frequently dysregulated in glioblastoma and is associated with tumor progression, making it a rational therapeutic target.

**Materials and Methods**

7. **Page 4, “CCK-8 Assay”: Specify exact duration of anethole treatment.**
   ✔ The duration has been specified as 24 hours, in line with Contant et al., 2021 (now cited in the revised manuscript).
8. **Page 5, “AO/EB Staining”: Clarify quantification method and cite reference.**
   ✔ We now state that apoptotic, necrotic, and viable cells were quantified using manual cell counting under a fluorescence microscope based on morphological and staining criteria, referencing Ribble et al., 2005.
9. **Page 6, “Molecular Docking”: Justify use of AutoDock over newer methods.**
   ✔ We have included a rationale noting that AutoDock remains a validated and widely used tool, particularly suitable for flexible ligand docking and preliminary screening of natural products due to its robust scoring functions and availability.
10. **Page 7, “Western Blot”: Were densitometry results normalized?**
    ✔ Yes. We now clarify that ImageJ software (NIH) was used for densitometry, and expression levels were normalized to β-actin as the loading control.

**Results**

11. **Page 8, Figure 1: Include sample size and SD for IC₅₀.**
    ✔ This information has been added: IC₅₀ values were derived from three independent experiments and are expressed as mean ± SD.
12. **Page 9, Apoptosis: Was flow cytometry used?**
    ✔ No flow cytometry was used. We clarify that apoptosis was assessed only via AO/EB staining in this study.
13. **Page 10, Docking: Was in vitro validation of target binding performed?**
    ✔ We now acknowledge that in vitro validation (e.g., via SPR or ITC) was not performed and have highlighted this as a limitation and future direction in the discussion.

**Discussion**

14. **Compare anethole with existing PI3K inhibitors like Wortmannin and LY294002.**
    ✔ We have added a paragraph discussing that while anethole demonstrates moderate affinity for PI3K, it may offer advantages over conventional inhibitors due to lower toxicity and broader multi-target effects. We also reference the known drawbacks of Wortmannin and LY294002 (e.g., instability, cytotoxicity).
15. **BBB permeability: Is there direct evidence for anethole crossing the BBB?**
    ✔ We clarify that while anethole is lipophilic and theoretically capable of BBB penetration, direct experimental evidence is lacking. We reference Yu et al., 2011 and recommend further pharmacokinetic studies.
16. **Limitations: No in vivo validation. Suggest adding statement.**
    ✔ We have included a clear statement acknowledging the lack of in vivo data and recommending future studies using animal models to validate the therapeutic potential and pharmacodynamics of anethole.

**Conclusion**

17. **Highlight future directions (e.g., combinational therapy, BBB studies).**
    ✔ The conclusion now includes suggestions for combinational therapy with current chemotherapeutics, investigation of BBB permeability, and in vivo validation as future directions.

**Reviewer #2**

1. **Compare MD-predicted binding constants with experimental data (ITC, BLI, SPR).**
   ✔ We appreciate this important suggestion. In the discussion, we have noted that while molecular docking predicted strong binding to PI3K, experimental confirmation using ITC, BLI, or SPR is necessary to validate these findings. This limitation has been acknowledged, and we propose these methods as next steps in validating the binding mechanism.

**Response to Editor’s Comments**

We thank the Editor for the valuable suggestions aimed at enhancing the clarity, consistency, and compliance of our manuscript with *PLOS ONE* guidelines. Below, we provide point-by-point responses to each of the recommendations and describe the revisions made accordingly:

**1. Adherence to Journal Guidelines**

- **Correct formatting of the references**
  **Response:** The entire reference list has been reformatted to align with the *PLOS ONE* style, as per the journal’s formatting requirements.
- **Consistency in the use of terminology and abbreviations**
  **Response:** All abbreviations and scientific terms have been reviewed for consistent usage throughout the manuscript. A list of abbreviations has also been provided where necessary.
- **Proper structuring of headings and subheadings**
  **Response:** The manuscript has been revised to ensure uniform formatting of all section headings and subheadings in accordance with the journal’s structure.

**2. Language and Clarity**

- **Grammatical and syntactical accuracy**
  **Response:** The manuscript has undergone a detailed language review to improve clarity, correct grammatical issues, and enhance the overall readability of the text.

**3. Methodology Section**

- **Additional details on source, purity, solvents, and quality control of anethole**
  **Response:** Anethole (99% purity) was obtained from Sigma-Aldrich (Oakville, ON, Canada) and dissolved in methanol to prepare a 3 mM stock solution. It was subsequently used at different concentrations for the experiments

**4. Figures and Tables**

- **Clear labeling, defined axes, and detailed captions**
  **Response:** All figures and tables have been reviewed and revised. Axes are now clearly labeled, units are defined, and figure captions have been expanded to fully describe the contents. Each figure and table is now properly referenced within the manuscript.

**5. Supplementary Data**

**6. Consistency in Presentation of Data**

- **Statistical analysis and experimental result presentation**
  **Response:** The manuscript has been carefully reviewed to ensure consistent reporting of sample size (n), standard deviations (SD), and p-values. All statistical analyses are now clearly defined in both the Methods and Results sections.

**7. Original Images for Blot/Gel Data**

- **Compliance with blot and gel image requirements**
  **Response:** As per *PLOS ONE*’s latest guidelines, the original, uncropped, and unadjusted image data underlying all Western blot figures have been included as Supporting Information files. We have also ensured that the preparation of these images and figure panels adheres to the specific requirements detailed in the journal’s policy.
