# Supplementary material for: Anethole inhibits human U87 Glioma cell proliferation by inducing apoptosis via the PI3K/AKT pathway
Source: PLoS One. 2025 Nov 21;20(11):e0336975. doi: 10.1371/journal.pone.0336975 (PMC12637905; doi:10.1371/journal.pone.0336975)
Supplement: Attachment — Submitted filename: Response_to_Reviewers_auresp_2.docx [file pone.0336975.s006.docx]

Dear Editor,

We thank you for the consideration of our manuscript. We also thank the reviewers for the suggestions for further strengthening of our manuscript and we have incorporated all suggestions of the respected reviewer.

Reviewer #3: Although the authors have been responsive, the following high-level suggestions may further strengthen the study:

Add a Note on Flow Cytometry as Future Work

While the authors clarified they did not use flow cytometry, suggesting its inclusion in future validation would demonstrate deeper awareness of methodological robustness in apoptosis detection.

Response: We appreciate this insightful recommendation. We have revised the Discussion section to acknowledge the value of incorporating flow cytometry (e.g., Annexin V/PI staining) in future studies for quantitative apoptosis detection.

Clarify Lipophilicity Measurement

The statement that anethole is “lipophilic” and may cross the BBB is speculative. Including a brief reference to LogP or in silico BBB permeability prediction (e.g., via SwissADME) would lend more support.

Response: We agree with the reviewer and have now included in silico SwissADME predictions in the Results and Discussion sections. These predictions show a consensus LogP of 2.79 and classify anethole as BBB permeant, supporting its CNS bioavailability (Supplementary Figure S1 and S2).

Densitometry Quantification – Loading Control Uniformity

Western blot images (if reviewed) should show uniform β-actin levels across conditions. While the use of ImageJ is stated, it's critical that raw blot bands (provided as supplementary) confirm integrity.

Response: We fully agree and have included densitometery quantification in Figure 2C, 2D, 5D-G. The uncropped raw Western blot images as Supplementary file, clearly showing consistent β-actin expression across all experimental conditions. This addition supports the integrity of protein loading and quantification.
