# Supplementary material for: Anethole inhibits human U87 Glioma cell proliferation by inducing apoptosis via the PI3K/AKT pathway
Source: PLoS One. 2025 Nov 21;20(11):e0336975. doi: 10.1371/journal.pone.0336975 (PMC12637905; doi:10.1371/journal.pone.0336975)
Supplement: Attachment — Submitted filename: Response_to_Reviewers_auresp_3.docx [file pone.0336975.s007.docx]

**Dear Editor**

We sincerely thank you and the anonymous reviewers for their constructive comments and valuable suggestions, which have significantly improved the quality and clarity of our manuscript. We have carefully addressed all concerns point by point, as detailed below. All textual changes have been incorporated into the revised version of manuscript and have been highlighted in **red**.

**Reviewer #4**

**This manuscript studies the anticancer cell effect of anethole in glioma cell lines and normal human astrocytes. Anethole has been broadly described in several cancer cell types, by different groups and being reviewed, showing antiproliferative and antimetastatic effect on neoplastic cells. As described by others, anethole induces apoptosis, cell cycle arrest, autophagy, antioxidant GHS, reduction of ROS, and metalloproteinases, etc., by interfering in different signaling pathways. The manuscript by Al Alwadh et al., demonstrates that anethole exerts the same effect in glioma cells as in other cancer cells, inducing apoptosis and reduction of PI3K/AKT phosphorylation. The mayor hypothesis and aim of this manuscript is to show the blocking effect of anethole in the PI3K/AKT pathway. for that, the authors analyzed in silico prediction of PI3K and anethole interaction. Although this is an interesting subject, the work should be completed by correcting some errors and adding some data as follows indicated:**

**Main concerns**

**1- The most interesting data is the predictable interaction of anethole and PI3k, some aspects in this regard should be considered. First if the effect of anethole takes place through interaction with PI3K and its inhibition, why does it not affect Normal human astrocytes (NHA)? The authors demonstrate, as previously described in cancer cells, reduction of PI3K and AKT phosphorylation only in one line of cancer cells. Then, the effect of anethole in NHA cells, as new data, would be nice to add in. Second, independent of the result about the effect in NHA, Anethole's stronger effect on cancer cells needs discussion. Could this be due to increased diffusion through cancer cell membranes? It would be interesting to discuss this, as increased anethole affinity to PI3K in cancer cells is unlikely. Finaly, AKT can take place through an independent mechanism as well, and it will be interesting to show either some downstream signal, or upstream signal that demonstrate PI3K direct inhibition.**

**Response:** We thank the reviewer for this insightful comment. While we acknowledge that inclusion of NHA apoptosis and phosphorylation data would strengthen the study, new wet-lab experiments are beyond the scope of this revision. To address this point, we have added a detailed explanation in the **Discussion section** discussing (i) why normal astrocytes show limited PI3K/AKT response, (ii) the potential role of altered membrane permeability and PI3K/AKT hyperactivation in glioma cells as the cause of selective sensitivity, and (iii) possible AKT-independent mechanisms requiring future validation. This clarification provides a mechanistic rationale for cancer-selective action of anethole and outlines future directions for in vivo and molecular validation.

**2- In discussion section, it is indicated “In addition, anethole has also been shown to elevate ROS accretion in cancerous cells, promoting oxidative stress and apoptosis [8]. ROS accumulation has been estimated to impair mitochondrial membrane potential, thereby activating intrinsic apoptotic sequences as described in breast cancer cells [26]”, the reference 8 showed that anethole reduces ROS and increased GSH antioxidant. Therefore, this paragraph and the argument should be changed accordingly.**

**Response:** We appreciate this correction. The cited paragraph has been revised in the **Discussion section** to accurately reflect the findings of reference [8], clarifying that anethole predominantly *reduces ROS levels and enhances GSH antioxidant capacity*, rather than increasing ROS. The interpretation was rewritten accordingly.

**3- Regarding the treatment with anethole, the most limiting aspect is that, even when anethole can diffuse through the BBB, due to low solubility in water and high elimination when orally administrated, the effect in gliomas looks limited. Some discussion also in this area should be nice to discuss.**

**Response:** We agree with the reviewer’s important observation. Additional discussion has been incorporated in the **Discussion section** addressing pharmacokinetic limitations of anethole such as low aqueous solubility and high first-pass metabolism. We have also added potential strategies—such as lipid-based nanoformulations, prodrug development, and intranasal delivery—to improve systemic and brain bioavailability.

**4- The figure legends need to indicate clearly whether the graphs show the media plus SD of an independent out of three experiments, or the media plus SD of three independent experiments.**

**Response:** We have revised all **figure legends** to explicitly state that “data are presented as mean ± SD of three independent biological experiments,” clarifying that each represents the mean of three independent replicates.

**5- The AO/EB analysis is used to indicate early and late apoptosis, the quantification of cells in these two statuses should be indicated in the graph. On the other hand, it would be convenient to show cell cycles of treated cells, also in NHA cells.**

**Response:** We appreciate this valuable suggestion. Quantitative analysis of total apoptosis (early + late) was added in the **Methods (AO/EB assay)** and described in **Figure 2 legend**. As additional flow cytometry experiments were not performed, we clarified in the **Discussion** that detailed early/late apoptosis and NHA apoptosis assays will be considered in future work.

**6- In general, it is indicated that experiments were performed three times, but whether the graph represents one out three or the media plus SD of three independent experiments need to be indicated.**

**Response:** This point has been addressed throughout the **Results** and **Figure legends**, which now specify that all data represent the *mean ± SD of three independent biological experiments*.

**7- The apoptosis assay needs to be performed with normal NHA cells, to consistently confirm the specific effect of anethole in gliomas. Also, the**

**Response:** We acknowledge the reviewer’s suggestion. While inclusion of NHA apoptosis data would provide valuable confirmation, conducting new experiments exceeds the current revision scope. However, this limitation is now explicitly stated in the **Discussion (last paragraph)**, and future studies will incorporate apoptosis assays on NHA and in vivo models to verify selectivity.

**8- The relative expression of p-PI3K and p-AKT needs to be calculated according to the total amount of PI3K and AKT protein, rather than to actine, and needs to be indicated in the Figure 5 legend.**

**Response:** We appreciate this important observation. As suggested, the densitometric analysis was recalculated and normalized to the respective total proteins (p-PI3K/PI3K and p-AKT/AKT). This correction has been clearly indicated in both the **Methods (Western blot)** and **Figure 5 legend**.

**Minor concerns**

**1- Material, please indicate the precedence of: cell lines, NHA cells, CCK-8 assay, and al used material in the material and methods section.**
**Response:** The **Materials and Methods** section was expanded to include the source, catalog numbers, and vendors for all reagents and cell lines (Section 2.1).

**2- Please, indicate the meaning of acronyms first time used in the text**
**Response:** All acronyms (e.g., NHA, AO/EB, TPSA, BBB) are now defined at first mention throughout the manuscript.

**3- Indication of the antibody clone used, and the company of precedence needs to be included in the material and methods section.**
**Response:** The **Western blot** subsection (2.7) now lists the antibody source, catalog number, and manufacturer for each primary antibody.

**4- Correct the sentence at page 35- “Cells were subsequently lysing was done using RIPA buffer”**
**Response:** The sentence has been corrected to “Cells were lysed using RIPA buffer.”

**5- Either Figures 5a and 5b have been mixed up, or the text in the legends and in the 'Results' section (3.4) is incorrect.**
**Response:** Thank you for noticing this. The mix-up was corrected—the figure panels and legends now correctly correspond to PI3K (Figure 5A) and JAK2 (Figure 5B).

**6- The images in Figures 3, 4, 5a and 5b should be clearer.**
**Response:** We increased the resolution of Figures 3–5

**Reviewer #5**

**1- Mechanistic Depth and Specificity: The study compellingly shows that anethole inhibits the PI3K/AKT pathway and induces apoptosis. However, to strengthen the claim that apoptosis is directly caused by PI3K/AKT inhibition (and not a parallel event), a rescue experiment would be highly valuable. Could the pro-apoptotic and anti-proliferative effects of anethole be reversed by introducing a constitutively active form of AKT (e.g., via plasmid transfection) into the U87 cells prior to anethole treatment? If such an experiment is beyond the scope of this revision, explicitly stating this as a key limitation and a requirement for future validation would strengthen the manuscript's conclusions. Where to place this: In the Discussion (to frame the findings) and/or the Conclusion (as a future direction).**

**Response:** We appreciate this valuable suggestion. As additional transfection experiments could not be performed at this stage, we have explicitly included this as a **key limitation** in the **Discussion (final paragraph)** and highlighted it in the **Conclusion** as a future research direction.

**2- The figures (e.g., Figure 2B, 5C) show representative Western blot bands, but the molecular weight markers are not visible. Including a lane with markers in the main figure or the supplementary uncropped blots is essential for verifying the identity of the protein bands. Figure 5A and 5B captions are swapped. Figure 5A describes the JAK2 interaction, while the image is labeled for PI3K, and vice versa. This must be corrected.**

**Response:** We have now included **uncropped blots with visible molecular weight markers** as supplementary material and corrected the swap between **Figure 5A and 5B** to align with the text.

**3- Statistical Analysis Description: The Methods section (2.9) states that experiments were performed in triplicate and data is presented as mean ± SD. For the colony formation assay, it would be helpful to specify how many technical replicates (wells) and biological replicates (independent experiments) were performed, as this assay typically has fewer replicates.**

**Response:** We agree and have updated the **Colony Formation Assay (Section 2.4)** to indicate that each condition included two technical replicates and three independent biological experiments.

**4- Language and Flow Minor Revisions: The manuscript is well-written but would benefit from a final proofread for minor grammatical redundancies. For example, in the Introduction (page 33): "...impede growth via cell cycle arrest in prostate cancer cells via arresting cell cycle." The phrase "via arresting cell cycle" is redundant and can be removed.**

**Response:** We thank the reviewer for this observation. The entire manuscript has been carefully proofread to correct redundancies and improve fluency. The specific phrase mentioned has been revised accordingly in the **Introduction**.

**We are deeply grateful to both reviewers for their thoughtful input, which helped us strengthen the manuscript. We hope the revisions satisfactorily address all concerns.**

**Sincerely,**
Dr. Mohammed Merae Alshahrani (Corresponding Author)
Department of Clinical Laboratory Sciences
Najran University, Saudi Arabia
Email: mmalshahrani@nu.edu.sa
